# Closing Editorial for the Special Issue: “Male Sexual and Reproductive Health: Clinical Aspects, Metabolic Profile, Environmental Factors and Lifestyle—Current Advances and Future Directions”

**DOI:** 10.3390/jcm14103342

**Published:** 2025-05-12

**Authors:** Fernando Mazzilli

**Affiliations:** AIED Center for Reproductive Medicine, Via Toscana 30, 00185 Rome, Italy; fernando.mazzilli@fondazione.uniroma1.it

## 1. Introduction

In recent years, significant advancements have been made in understanding male reproductive health and sexual function. This Editorial summarizes the findings of this Special Issue and other recent studies, highlighting interesting discoveries and identifying persistent gaps in the literature.

## 2. Erectile Dysfunction

Erectile dysfunction (ED) is defined as “recurrent and persistent inability, partial or complete, to achieve or maintain an erection firm enough for satisfactory sexual intercourse in the presence of proper erotic stimuli” [1,2]. The causes of ED can be distinguished as occurring alongside male hypoactive sexual desire disorder or alongside normoactive sexual desire, where the former can be related to psychogenic conditions or organic diseases [3,4].

In our first contribution, Wroblewski et al. demonstrate a strong interdependence between mental and physical health in post-myocardial infarction (MI) patients, with ED being a key factor affecting self-esteem.

Modern behaviors, including internet addiction, social media use, and online pornography, have also been shown to have significant effects on male sexual health. According to Pawlikowska-Gorzelańczyk et al. (Contribution 2), social media addiction had a negative impact on International Index of Erectile Function (IIEF) scores, whereas general pornography use had no impact on men’s sexual health. However, more extensive use of pornography was correlated with lower IIEF scores. The Authors also observed that social media addiction negatively affected men’s sexual functioning during the COVID-19 pandemic.

For therapeutic aspect, many studies are being conducted with the aim of expanding current therapeutic availability and seeking alternatives for the resolution of sexual dysfunctions. Trifu et al., in Contribution 3, compared intra-meatal tadalafil cream to oral administration, offering a novel approach with potential benefits for patient compliance and reduced side effects.

## 3. Infertility and Genetics

Infertility, a disease of the male or female reproductive system, is defined by the failure to achieve pregnancy after at least 12 months of regular unprotected sexual intercourse [5]. Despite a thorough diagnostic approach, the cause of infertility still remains unknown in a large percentage of male partners in infertile couples.

Among the various causes studied (congenital and acquired, pre-pubertal and post-pubertal, etc.), numerous studies have been carried out on the effects of oxidative stress on spermatozoa. Normally, the spermatozoa produce a small amount of reactive oxygen species (ROS), which play an important role during the acrosomal reaction necessary for oocyte penetration. However, ROS overproduction or a deficit of the “scavenger system” (superoxide anion, H_2_O_2_, glutathione) can occur. Subsequent oxidative stress induces peroxidation of the spermatic membrane, and therefore a defective sperm function [6].

Further, Chen et al. (Contribution 4) evaluated the impact of oxidative stress on sperm quality, even in cases without overt infertility, emphasizing the need for further molecular research.

Genetic factors have emerged as being increasingly important in the diagnosis of male infertility [7,8]. Their importance lies in the ability of new Assisted Reproduction Techniques (ARTs), such as Testicular Sperm Aspiration (TESA) and Testicular Sperm Extraction (TESE), to allow for fertilization even in cases of cryptozoospermia or obstructive azoospermia [9,10].

Advocating for genetic screening and personalized medicine, Mazzilli et al. (Contribution 5) explored the role of genetic causes in male factor infertility (including karyotype, the *CFTR* gene mutation plus variant of the IVS8-5T polymorphic trait, Y chromosome microdeletion, and the use of next-generation sequencing panels to analyze the genes implicated in Congenital Hypogonadotropic Hypogonadism).

Finally, the study by Lahimer et al. (Contribution 6) highlighted the association between paternal age and alterations in sperm parameters, DNA integrity, and methylation profiles. This work emphasized how aging impacts male fertility and emphasizes the importance of early intervention and counseling in family planning.

## 4. Lifestyle

Lifestyle factors (i.e., diet, physical activity, smoking, alcohol consumption, drug use, and stress) certainly play a critical role in both sexual and reproductive function [11].

Viken et al. (Contribution 7) revealed that physical fitness, and muscle strength in particular, is closely correlated with sexual function, reinforcing the importance of exercise and physical activity for overall well-being.

On the other hand, Skrzypiec-Spring et al. (Contribution 8) documented the adverse effects of anabolic–androgenic steroid misuse among amateur athletes, highlighting the need for public health interventions to vary this behavior.

With regard to the reproductive function front, the delicate balance between intense physical activity and reproductive health is highlighted by Greco et al. (Contribution 9), who synthesized evidence on the effects of training regimens on semen parameters in athletes. Intensive training could worsen seminal parameters and, consequently, male fertility. Therefore, their findings suggested the use of tailored training programs that mediate both performance and fertility.

Finally, Barbagallo et al. (Contribution 10) highlighted the detrimental impact of smoking on male infertility, demonstrating how nicotinic acetylcholine receptors influence testicular function and emphasizing the importance of stopping smoking.

Therefore, there is a pressing need to develop targeted interventions that address the behavioral and lifestyle factors influencing male reproductive and sexual health.

## 5. Prostate

Recent research has also focused on prostate health. Studies have revealed a significant correlation between lower urinary tract symptoms (LUTSs) and ED, particularly in men over 50 years of age [12]. Porav-Hodade et al. (Contribution 11) found that prostate length, rather than overall size, may play a significant role in lower urinary tract symptoms, as well as in ED, providing further insight into prostate-related pathologies.

On the pharmacological front, Yanagida et al. (Contribution 12) introduced vibegron, a β3-adrenoceptor agonist for treating persistent overactive bladder (OAB) symptoms, as an add-on therapy for promising treatment of benign prostatic hyperplasia, showing improvements in both urinary symptoms and intercourse satisfaction.

## 6. Conclusions

While these studies provide valuable insights, there are still significant knowledge gaps in the literature. The molecular mechanisms underlying many of these findings require further elucidation. Longitudinal studies are needed to establish causality, and a multidisciplinary approach integrating genetics, endocrinology, psychology, and public health is essential for gaining a comprehensive understanding.

Therefore, scientific research is required to address unresolved questions and translate these insights into effective clinical strategies.
